# Migration Safety of Perfluoroalkyl Substances from Sugarcane Pulp Tableware: Residue Analysis and Takeout Simulation Study

**DOI:** 10.3390/molecules30153166

**Published:** 2025-07-29

**Authors:** Ling Chen, Changying Hu, Zhiwei Wang

**Affiliations:** 1 Key Laboratory of Product Packaging and Logistics of Guangdong Higher Education Institutes, College of Packaging Engineering, Jinan University, Zhuhai 519070, China; chenlingwh@stu2022.jnu.edu.cn; 2 Department of Food Science and Engineering, Jinan University, Guangzhou 510632, China

**Keywords:** sugarcane pulp tableware, per- and polyfluoroalkyl substances (PFASs), residue, migration, food contact material, takeout simulation

## Abstract

The rapid growth of plant-based biodegradable tableware, driven by plastic restrictions, necessitates rigorous safety assessments of potential chemical contaminants like per- and polyfluoroalkyl substances (PFASs). This study comprehensively evaluated PFAS contamination risks in commercial sugarcane pulp tableware, focusing on the residues of five target PFASs (PFOA, PFOS, PFNA, PFHxA, PFPeA) and their migration behavior under simulated use and takeout conditions. An analysis of 22 samples revealed elevated levels of total fluorine (TF: 33.7–163.6 mg/kg) exceeding the EU limit (50 mg/kg) in 31% of products. While sporadic PFOA residues surpassed the EU single compound limit (0.025 mg/kg) in 9% of samples (16.1–25.5 μg/kg), the levels of extractable organic fluorine (EOF: 4.9–17.4 mg/kg) and the low EOF/TF ratio (3.19–10.4%) indicated inorganic fluorides as the primary TF source. Critically, the migration of all target PFASs into food simulants (water, 4% acetic acid, 50% ethanol, 95% ethanol) under standardized use conditions was minimal (PFOA: 0.52–0.70 μg/kg; PFPeA: 0.54–0.63 μg/kg; others < LOQ). Even under aggressive simulated takeout scenarios (50 °C oscillation for 12 h + 12 h storage at 25 °C), PFOA migration reached only 0.99 ± 0.01 μg/kg in 95% ethanol. All migrated levels were substantially (>15-fold) below typical safety thresholds (e.g., 0.01 mg/kg). These findings demonstrate that, despite concerning residue levels in some products pointing to manufacturing contamination sources, migration during typical and even extended use scenarios poses negligible immediate consumer risk. This study underscores the need for stricter quality control targeting PFOA and inorganic fluoride inputs in sugarcane pulp tableware production.

## 1. Introduction

The global food service market, particularly in China, has experienced rapid growth in recent years. Accompanying this growth is a substantial consumption of disposable plastic tableware. Global plastic consumption reached 547 million tonnes in 2020, with China being the largest consumer [1]. In response to escalating environmental concerns, “plastic restriction orders” have been implemented worldwide, prompting the food service industry to seek sustainable alternatives to conventional plastics. Plant-based materials, such as those derived from sugarcane bagasse pulp, have emerged as promising substitutes due to their biodegradability and abundant raw material sources [2].

However, achieving adequate water and oil resistance in plant-based tableware often necessitates the addition of processing aids during manufacturing [3]. Per- and polyfluoroalkyl substances (PFASs), a large class of synthetic chemicals characterized by the replacement of all hydrogen atoms attached to carbon atoms in the alkyl chain with fluorine atoms (C-F bonds), are frequently employed for this purpose due to their exceptional surfactant properties [4,5]. PFASs can be categorized based on chain length: short-chain (C4–C7) and long-chain (C ≥ 8). Examples include perfluorooctanoic acid (PFOA), perfluorooctane sulfonic acid (PFOS), perfluorononanoic acid (PFNA), perfluorohexanoic acid (PFHxA), and perfluoropentanoic acid (PFPeA) [6]. Concerns regarding PFASs stem from their documented persistence, bioaccumulation potential, and adverse health effects, including carcinogenicity [7], reproductive toxicity [8], hepatotoxicity [9,10], and immune system disruption [11]. Their extreme environmental persistence has led to their designation as contaminants of global concern [12]. While short-chain PFASs were introduced as alternatives to regulated long-chain PFASs (e.g., PFOA, PFOS) due to their shorter estimated half-lives [9], emerging evidence suggests that they may also pose significant health risks, including hepatotoxicity [13]. Consequently, the global regulatory scrutiny of PFASs is intensifying. Notably, Japan has banned PFHxS and related substances since 2025, and the revised EU Packaging and Packaging Waste Directive (PPWD) sets limits for packaging materials: total fluorine (TF) ≤ 50 mg (F)/kg and individual PFAS ≤ 0.025 mg/kg [14].

Research on PFASs in food contact materials (FCMs) is expanding. Traditional targeted analytical methods identify only known compounds. To address potential unknown organofluorine, techniques like oxygen bomb combustion coupled with ion-selective electrode (ISE) or combustion ion chromatography (CIC) are employed to measure the levels of TF and EOF [15]. Targeted analysis using liquid chromatography–tandem mass spectrometry (LC-MS/MS) has been applied to study PFAS distribution in various FCMs like paper–plastic composites, plastic films, and trays [16], as well as in plant-based plates and oil-proof coated papers [17]. Nevertheless, data on fluorine distribution and specific PFAS contamination in novel sugarcane pulp tableware remain limited. Crucially, the safety risks associated with PFAS migration under real-world conditions, such as those encountered during takeout food delivery and storage, are poorly understood.

Therefore, this study aimed to perform the following: (1) Screen for potential PFAS contamination in commercially available sugarcane pulp tableware by measuring TF and EOF levels. (2) Quantify the residues of five specific PFASs (PFOA, PFOS, PFNA, PFHxA, PFPeA) using optimized LC-MS/MS methods. (3) Investigate the migration of these PFASs into food simulants representing aqueous, acidic, alcoholic, and fatty foods under standardized conditions. (4) Evaluate the impact of simulated takeout delivery (mechanical stress, temperature) and subsequent storage conditions on PFAS migration. Two primary methods exist: the targeted summation of known PFASs and non-targeted screening via mass spectrometry [18]. We selected targeted analysis due to its regulatory relevance for specific compounds (PFOA/PFOS), cost-effectiveness, and validated accuracy for low-concentration quantification in complex matrices. This comprehensive assessment provides critical data for evaluating the safety profile of sugarcane pulp tableware concerning PFAS exposure.

## 2. Results and Discussion

### 2.1. TF and EOF Content Analysis

#### 2.1.1. ISE Method Validation

The ISE method demonstrated good performance. Calibration curves (Y = 407.76 − 53.03X) for both TF and EOF showed excellent linearity (R^2^ = 0.9998) in the range of 4–100 μg/L (EOF) and 6–100 μg/L (TF). The limits of detection (LODs) and quantification (LOQs) were 2.0 μg/L and 4.0 μg/L for EOF and 3.0 μg/L and 6.0 μg/L for TF, respectively. Spike recovery tests at three levels (10, 40, 80 μg (F)/L) in a low-TF sample matrix yielded average recoveries of 87.1–111.4% for TF and 97.5–108.7% for EOF, with relative standard deviations (RSDs) ≤ 7.9%, meeting the requirements for quantitative analysis (Table 1 and Table 2). Expanded uncertainty: TF ± 5.2%(RSD), EOF ± 6.8%(RSD), propagated from weighing, dilution, and ISE measurement uncertainties.

#### 2.1.2. TF and EOF Content in Samples

**Figure 1:**
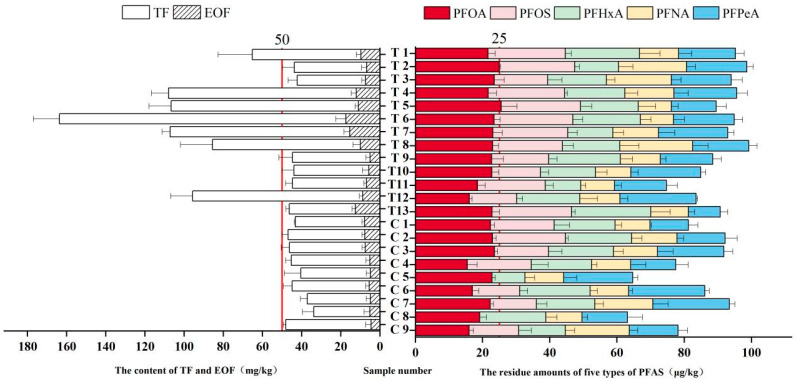
Distribution of TF, EOF, and residues of five PFASs in sugarcane pulp tableware samples (*n* = 22). Red lines indicate EU PPWD limits (TF: 50 mg/kg; single PFAS: 0.025 mg/kg). Error bars represent standard deviation.

The TF content in the 22 sugarcane pulp tableware samples ranged from 33.7 to 163.6 mg/kg (Figure 1). While China currently lacks specific TF limits for FCMs, 31% (7/22) of samples exceeded the EU PPWD limit of 50 mg/kg. EOF content ranged from 4.9 to 17.4 mg/kg. All samples complied with the Danish regulatory limit (20 mg/kg) and the Californian limit (100 mg/kg) for organic fluorine in food packaging [19,20]. The ratio of EOF to TF ranged from 3.19% to 10.4%, consistent with the findings by Schultes et al. [21] and suggesting that a significant portion of the total fluorine was inorganic or bound in non-extractable forms. The high TF exceedance rate highlights potential PFAS contamination concerns in some sugarcane pulp tableware products entering the market. The phenomenon of high TF levels but low EOF levels reflects the fact that TF encompasses a broader range of fluorine forms. When processing aids such as NaF (an inorganic fluoride) are present, they contribute significantly to TF. However, NaF itself is inorganic and will not be extracted in EOF analysis (as EOF is specifically designed for organic fluorine) [22], resulting in TF levels being significantly higher than EOF levels [23]. In artificial turf (AT) studies, TF was detected in all 51 samples (range: 16–661 μg F/g), whereas EOF and targeted PFASs were detected in fewer than 42% of samples (EOF < 200 ng F/g). Water extraction experiments confirmed that inorganic fluorides (e.g., fluoride) did not affect the results after removal, indicating that inorganic fluorine is a major component of TF but is not accounted for in EOF [24]. This directly supports the view that processing aids like NaF may dominate high TF levels, as NaF, being inorganic, is not included in EOF extraction. In dust samples, EOF levels accounted for less than 1% of TF, suggesting the presence of large amounts of unidentified fluorides (including potentially inorganic fluorine from processing aids), while targeted PFASs contributed at most 9% of EOF [25]. Similarly, in plastic and rubber products, TF values varied widely, but EOF was often below the limit of detection (LOD), highlighting the dominant role of inorganic fluorine in TF [22]. Low EOF/TF (3–10%) aligns with studies on food packaging [19,24], confirming inorganic fluorides (e.g., NaF) as the primary TF source. In plastic/rubber, EOF is often undetectable [22].

### 2.2. PFAS Residue Analysis

#### 2.2.1. LC-MS/MS Method Validation for Residues

The optimized LC-MS/MS method for PFAS residues exhibited excellent sensitivity and reliability. Otal ion chromatograms of five PFAS reference standards in LC-MS/MS see Supplementary Materials. Calibration curves were linear (R^2^ ≥ 0.9998) over the range of 0.5–40 μg/L for all five PFASs. LODs ranged from 0.2 to 0.5 μg/L, and LOQs ranged from 0.5 to 1.2 μg/L (Table 3). Spike recovery experiments (5, 7.5, 30 μg/L) in a low-PFAS sample matrix and methanol solvent yielded average recoveries of 80.0–120.0% with RSDs ≤ 8.9% (Table 4), demonstrating satisfactory accuracy and precision for residue quantification. PFOA, PFOS, and PFNA (C8–C9) were selected as regulated long-chain PFASs; PFHxA/PFPeA (C5–C6) represent common short-chain alternatives [6,14]. This covers >80% of detectable PFASs in FCMs [16]. Therefore, we only evaluated five per- and polyfluoroalkyl substances (PFASs).

#### 2.2.2. Optimization of PFAS Extraction

**Figure 2:**
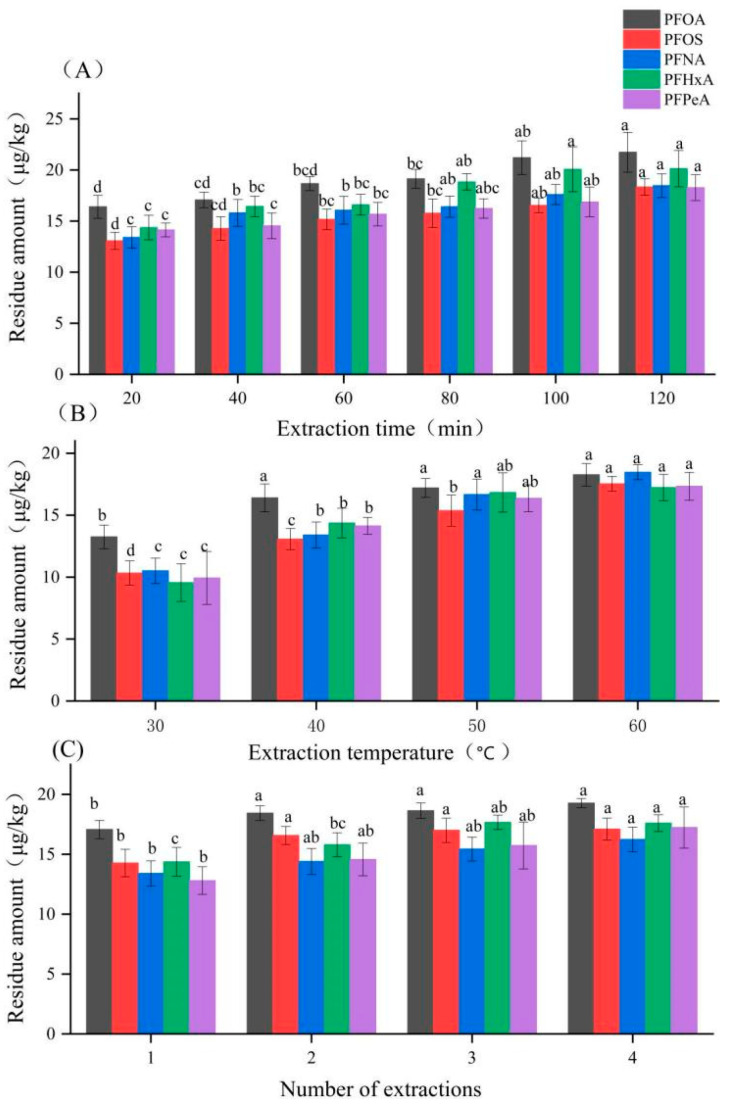
Effect of (**A**) extraction time, (**B**) extraction temperature, and (**C**) number of extraction cycles on recovery of five PFASs from sugarcane pulp tableware. Note: Different lowercase letters in the figure indicate statistically significant differences for the same type of sample across varying extraction times, temperatures, and extraction cycles; identical letters denote no significant difference.

Extraction efficiency was significantly influenced by time, temperature, and the number of cycles (Figure 2). Residue levels increased with longer extraction times and higher temperatures up to 60 °C and 100 min, beyond which no significant improvement was observed. Increasing the number of extraction cycles enhanced recovery, but a second extraction yielded minimal additional gain. Therefore, conducting ultrasonic extraction twice with preheated methanol (60 °C) for 100 min each was established as the optimal procedure.

#### 2.2.3. PFAS Residues in Samples

Residues of the five target PFASs were detected in all samples (Figure 1). PFOA residues ranged from 16.1 to 25.5 μg/kg (mean: 21.4 μg/kg), exceeding the EU PPWD/REACH single PFAS limit of 0.025 mg/kg (25 μg/kg) in 9% (2/22) of samples. PFOS residues ranged from 13.7 to 23.4 μg/kg (mean: 17.4 μg/kg). PFPeA residues ranged from 9.5 to 22.7 μg/kg (mean: 18.3 μg/kg). PFHxA residues ranged from 10.6 to 23.6 μg/kg (mean: 17.5 μg/kg). PFNA residues ranged from 9.8 to 21.6 μg/kg (mean: 13.4 μg/kg). All PFOS, PFNA, PFHxA, and PFPeA residues were below 25 μg/kg.

The PFOA exceedances could stem from the following: (1) The intentional addition of PFAS-based water/oil repellents during manufacturing [2]. (2) Contamination from PFASs in process water or the sugarcane pulp raw material itself [12,26,27,28]. (3) The degradation of precursor PFASs (e.g., fluorotelomer alcohols, polyfluoroalkyl phosphate diesters—diPAPs) present in the material or additives into PFOA [29,30] or into shorter-chain acids like PFPeA and PFHxA [31,32]. The slightly higher mean level of PFPeA compared to PFOS (18.3 vs. 17.4 μg/kg) aligns with the trend of decreasing PFOS usage and increasing reliance on alternatives, including shorter-chain compounds and precursors, as observed by Monge Brenes et al. [27]. PFPeA residues may derive from diPAP degradation [31], though oxidative conversion was not tested. See Liu et al. [29] for degradation pathways.

### 2.3. PFAS Migration Analysis

#### 2.3.1. LC-MS/MS Method Validation for Migration

The LC-MS/MS method was validated for quantifying migration into the four food simulants. Good linearity (R^2^ ≥ 0.9771) was achieved within the range of 0.5–40 μg/L for most analyte/simulant combinations. LODs ranged from 0.1 to 0.9 μg/L, and LOQs ranged from 0.3 to 3.0 μg/L (Table 5), demonstrating sufficient sensitivity for trace migration analysis. This study employed traditional solvent extraction methods for PFAS extraction, which are simple and rapid. Moreover, several novel green techniques have been applied to PFAS extraction. For instance, a grooved solid-phase microextraction (SPME) device utilizing a matrix-compatible coating (HLB-WAX/PAN) enables the direct extraction of PFASs from intact meat samples, exhibiting low matrix effects (−13.7% to 11.1%) and strong mechanical stability [33]. When coupled with LC-MS/MS, SPME can analyze EPA-regulated PFASs in water samples. The extraction conditions for different environmental matrices were optimized by studying the adsorption/desorption mechanisms of the ion-exchange extraction phase [34]. Compared to SPME, micro-solid-phase extraction (μSPE) technology demonstrates superior PFAS recovery rates in multiphase media [35].

#### 2.3.2. Migration into Food Simulants Under Intended Use Conditions

**Figure 3:**
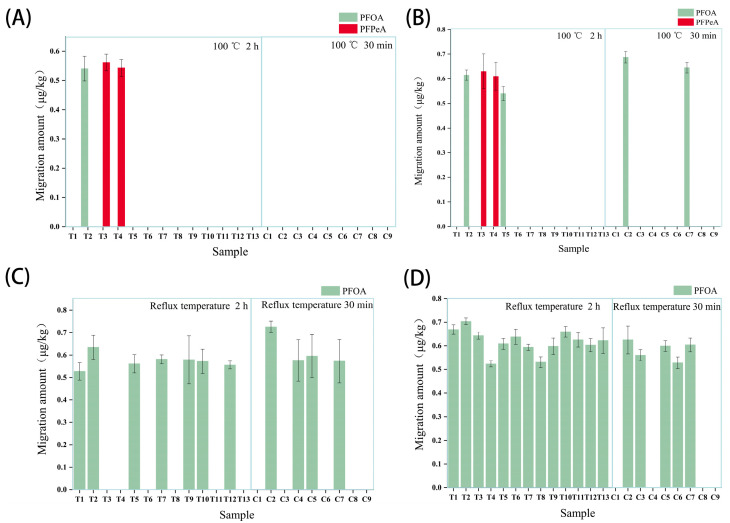
Migration levels of five PFASs from sugarcane pulp tableware into (**A**) ultrapure water, (**B**) 4% acetic acid, (**C**) 50% ethanol, and (**D**) 95% ethanol under intended use conditions. Values below LOQ are not shown (LOQs: PFOA/PFPeA ~0.5 μg/kg, PFOS/PFNA/PFHxA ~0.5–3 μg/kg). Dashed line indicates 0.01 mg/kg (10 μg/kg).

The migration value of PFNA, PFOS, and PFHxA into all four simulants under the standardized test conditions (Table 6) was below their respective LOQs. A trace migration of PFOA and PFPeA was detected (Figure 3). PFOA migration ranged from 0.52 to 0.70 μg/kg (mean across simulants: 0.62 μg/kg). PFPeA migration ranged from 0.54 to 0.63 μg/kg (mean: ~0.59 μg/kg). Critically, all measured migration values were well below the commonly referenced specific migration limit (SML) of 0.01 mg/kg (10 μg/kg) proposed for PFASs in some regulatory contexts or used for risk assessment. The max PFOA migration (0.7 μg/kg) is 0.001% of EFSA’s TDI (1.8 μg/kg bw/day). Cumulative exposure remains negligible. Sugarcane bagasse tableware may continuously release PFASs (per- and polyfluoroalkyl substances) during disposal processes (composting or landfilling). For instance, during composting, some long-chain PFASs (such as PFPeA) may be degraded or transformed, while short-chain PFASs (such as PFBS) may be newly formed or persist as residues [36]. Furthermore, if PFASs from compost enter agricultural systems through land application, they may form cyclical pollution via crop uptake and groundwater infiltration, increasing human exposure risks [37]. Additionally, PFAS-containing food packaging, like sugarcane bagasse tableware, under anaerobic decomposition conditions in landfills, releases volatile PFASs into the gaseous phase, contaminating the environment [38]. These hazards must not be overlooked. Future efforts require further in-depth studies on how to achieve the harmless treatment of such materials.

The higher detection frequency and slightly higher levels of PFOA in 50% and 95% ethanol compared to water and acetic acid can be attributed to its longer carbon chain length (C8 vs. C5 for PFPeA) and higher octanol–water partition coefficient (Log P ~3.6 for PFOA vs. ~1.98 for PFPeA), leading to increased affinity for the organic simulants [39]. The very low or non-detectable migration of PFOS, PFNA, and PFHxA aligns with their stronger expected partitioning to the solid phase (sediment/particulate matter) compared to PFOA and PFPeA in aqueous environments, as described by phase distribution models [40]. Shen et al. reported similar partitioning trends: PFPeA predominantly in water, PFOA near equilibrium, and longer-chain PFASs (PFOS, PFNA, PFHxA) favoring the solid phase [41]. Bamboo fiber tableware shows higher PFOS migration values (2.1 μg/kg) [20], while PLA exhibits lower TF levels (<20 mg/kg) [40]. This explains their minimal migration observed here.

#### 2.3.3. Effect of Oscillation Time on PFOA Migration

**Figure 4:**
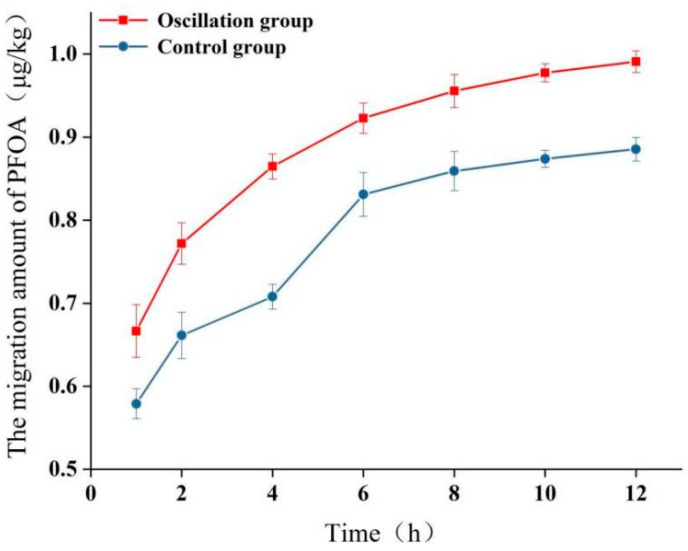
Effect of oscillation time (120 rpm) at 50 °C on migration of PFOA into 95% ethanol. Static condition at 50 °C is shown for comparison. Error bars represent standard deviation (*n* = 3).

Figure 4 shows the migration of PFOA into 95% ethanol under static and oscillating conditions at 50 °C over 12 h. Under static conditions, migration increased gradually, reaching 0.88 ± 0.02 μg/kg at 12 h. Oscillation significantly enhanced migration kinetics; migration levels were consistently higher than those under static conditions at each time point. After 12 h of oscillation, PFOA migration reached 0.99 ± 0.01 μg/kg, approximately 1.1 times higher than that for the static control at 12 h. While the absolute increase is small, this demonstrates that mechanical agitation during transport can accelerate the migration process. Nevertheless, even after prolonged agitation, the migration level remained far below 0.01 mg/kg. It is important to note that this study focused on five specific PFASs; the cumulative migration of other PFASs present in the material could be higher. The enhancement in oscillatory transport (such as fluctuations in transport rates or the emergence of concentration peaks) is primarily attributed to nonlinear adsorption processes and interfacial competition effects [42]. When PFASs migrate into the environment, their adsorption behavior is not a simple linear partitioning but is strongly influenced by factors such as concentration, PFAS molecular structure, and environmental conditions (e.g., pH, ionic strength) [42]. This leads to an oscillatory enhancement in PFAS transport rates under varying concentration gradients—for instance, faster migration at low concentrations but slower migration at high concentrations due to adsorption saturation [43]. Although oscillatory transport exhibits localized enhancement, the absolute overall transport level remains low [44]. The main reason for this is that PFASs demonstrate high adsorption affinity in the matrix, causing most PFASs to be immobilized at adsorption sites, significantly retarding migration [45,46]. Moreover, the high porosity of sugarcane pulp may accelerate migration vs. denser materials [45].

#### 2.3.4. Effect of Storage Conditions on PFOA Migration

**Figure 5:**
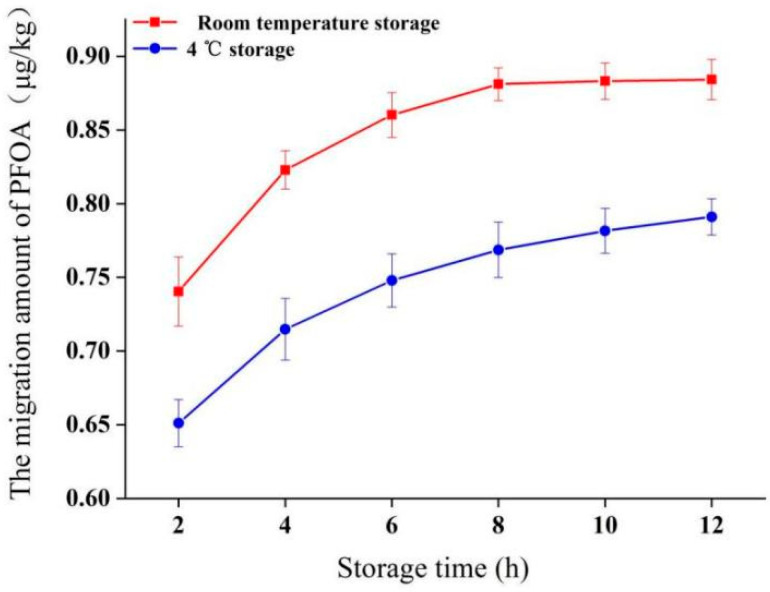
Migration of PFOA into 95% ethanol during storage at 4 °C or 25 °C after 1 h of oscillation (120 rpm) at 50 °C (simulating takeout delivery). Migration level immediately after oscillation (0 h storage) is shown. Error bars represent standard deviation (*n* = 3).

Following 1 h of simulated transport (oscillation at 50 °C), samples were stored for up to 12 h at 4 °C or 25 °C (Figure 5). Migration continued during storage, with slightly higher levels observed at room temperature compared to refrigeration. After 12 h of storage at 25 °C, PFOA migration was 0.88 ± 0.01 μg/kg, approximately 1.1 times higher than the level after 12 h at 4 °C (0.79 ± 0.01 μg/kg). Simulated reheating in hot water after storage did not result in a significant additional release of PFOA from the tested sample into the water simulant. Crucially, under all tested storage scenarios, PFOA migration remained significantly below 0.01 mg/kg.

## 3. Materials and Methods

### 3.1. Materials and Reagents

Samples: A total of 22 commercially available sugarcane pulp tableware items, comprising 13 food containers (T1–T13) and 9 tea cups (C1–C9), were purchased from online retail platforms. Key specifications (maximum use temperature, microwave safety claim) are summarized in Table 7. Samples were cleaned with ultrapure water and dried at room temperature before analysis.

Reagents and Standards: Fluoride ion standard solution (1000 mg/L, Shanghai Macklin Biochemical Co., Ltd., Shanghai, China); Sodium carbonate anhydrous (Analytical Reagent, AR, 99.5%), Sodium bicarbonate (AR, ≥99.8%), Sodium hydroxide (AR, 96%), Trisodium citrate dihydrate (AR, 99.0%), Sodium chloride (AR, 99.5%), Ammonium acetate (HPLC grade, 99.0%) (Aladdin Biochemical Technology Co., Ltd., Shanghai, China); Glacial acetic acid, Methanol (HPLC grade), Ethanol (HPLC grade), Acetonitrile (HPLC grade) (Shanghai Macklin Biochemical Technology Co., Ltd., Shanghai, China); PFOS, PFOA, PFNA, PFHxA, PFPeA standard solutions in methanol (100 mg/L) (ANPEL Laboratory Technologies Inc., Shanghai, China). Ultrapure water (resistivity ≥ 18.2 MΩ·cm) was generated by a UPTC-10 system (Shanghai Lichen Bangxi Instrument Technology Co., Ltd., Shanghai, China).

### 3.2. Instrumentation and Equipment

Oxygen bomb combustion system (YCY-4 Oxygen Filling Device, Oxygen Bomb Combustion Vessel, Nanjing Sangli Electronic Equipment Factory, Nanjing, China). Ion Meter (PXSJ-216F, Shanghai Yidian Analysis Instrument Co., Ltd., Shanghai, China) with Fluoride Ion Selective Electrode (PF-2-01), Calomel Reference Electrode (232-01), and Temperature Electrode (T-818-Q). Electronic Balance (AR224CN, OHAUS Instruments Co., Ltd., Shanghai, China). Ultrasonic Cleaner (KQ5200DE, Kunshan Ultrasonic Instrument Co., Ltd., Suzhou, China). Ultra-Pure Water System (UPTC 10, Shanghai Lichen Bangxi Instrument Technology Co., Ltd.). Digital Display Electric Hot Plate (DB-2EFS, Shanghai Lichen West Instrument Technology Co., Ltd., Shanghai, China). LC-MS/MS System (SCIEX Triple Quad 4500, AB Sciex, Framingham, MA, USA). Thermostatic Drying Oven (DHG-9140A, Shanghai Jinghong Laboratory Equipment Co., Ltd., Shanghai, China). Constant Temperature Water Bath Shaker (SHA-CA, Changzhou Aohua Instrument Co., Ltd., Changzhou, China).

### 3.3. Determination of TF and EOF Content

Reagents were prepared according to standard protocols: 6 mol/L NaOH; a total ionic strength adjustment buffer (pH 5–6) containing glacial acetic acid, NaCl and disodium citrate dihydrate; a fluoride absorption solution composed of Na_2_CO_3_ and NaHCO_3_; and a series of perfluorooctanoic acid (PFOA) working standards at 10, 40, and 80 µg F/L, obtained by the serial dilution of the stock solution, for recovery validation.

Total fluorine (TF). Accurately weighed subsamples (0.20 g, finely cut) were transferred to combustion crucibles. Ten milliliters of the fluoride absorption solution was placed in an oxygen flask apparatus; after sealing, the headspace was pressurized with O_2_ to 2.0 MPa. Combustion products were trapped in the absorption solution and an additional 20 mL of ultrapure water. Each batch included three replicates and one procedural blank.

Extractable organofluorine (EOF). Subsamples (0.50 g) were extracted with 10 mL ethanol under ultrasonication for 30 min. Eight milliliters of the extract was taken to near dryness under a gentle nitrogen stream and reconstituted in 1.5 mL ethanol. The concentrated solution was transferred to a crucible and submitted to the same oxygen flask combustion and absorption protocol as described for TF [18,47]. Triplicate analyses were performed.

An aliquot (20 mL) of the sample solution (TF or EOF absorption solution) was transferred to a 50 mL volumetric flask. TISAB solution (20 mL) was added, and the volume was made up to the mark with ultrapure water. The solution was poured into a clean PP beaker, and the fluoride ion concentration was measured using an ion meter equipped with the fluoride ISE and reference electrode. Calibration was performed using standard solutions prepared in the appropriate matrix [48].

$$\mathrm{TF}\;\mathrm{Content}\;(C_{1},\;\mathrm{mg}/\mathrm{kg}):\;C_{1}=\frac{(C_{i}-C_{0})\times V\times a}{1000\times m}$$

In this formula, *C*_1_ is the TF content in the sample, mg/kg; *C_i_* is the fluoride ion concentration in the test solution, μg/L; *C*_0_ is the fluoride ion concentration in the blank solution, μg/L; *a* is the dilution factor; *V* is the constant volume, mL; 1000 is the unit conversion factor; *m* is the sample weight, g.

$$\mathrm{EOF}\;\mathrm{Content}\;(C_{2},\;\mathrm{mg}/\mathrm{kg}):\;C_{2}=\frac{(C_{i}-C_{0})\times V\times a}{1000\times 0.8\times m}$$

In this formula, *C*_2_ is the EOF content in the sample, mg/kg; *C_i_* is the fluoride ion concentration in the test solution, μg/L; *C*_0_ is the fluoride ion concentration in the blank solution, μg/L; *a* is the dilution factor; *V* is the constant volume, mL; 1000 is the unit conversion factor; 0.8 is the result correction factor; *m* is the sample weight, g.

### 3.4. Determination of PFAS Residues

#### 3.4.1. Optimization of Extraction Procedure

The extraction efficiency for the five PFASs from the sugarcane pulp matrix was optimized by varying the extraction temperature (30 °C, 40 °C, 50 °C, 60 °C), extraction time (20, 40, 60, 80, 100, 120 min), and number of extraction cycles (1, 2, 3, 4). Methanol was used as the extraction solvent. The extraction temperature was limited to 60 °C due to the boiling point of methanol.

#### 3.4.2. Sample Extraction for Residue Analysis

Sample pieces (0.10 g ± 0.1 mg) were weighed into a 10 mL PP centrifuge tube. Preheated methanol (5 mL, 60 °C) was added. The mixture was ultrasonicated at 60 °C for 100 min. After centrifugation at 12,000 rpm, 10 min, the supernatant was transferred to a clean PP tube. The extraction was repeated once with fresh methanol (5 mL, 60 °C, 100 min ultrasonication). The combined extracts were concentrated to near dryness under nitrogen at 40 °C. The residue was reconstituted in 1 mL methanol, vortex-mixed, filtered through a 0.22 μm nylon syringe filter into an LC vial, and stored at 4 °C until LC-MS/MS analysis. Triplicate analyses were conducted.

#### 3.4.3. LC-MS/MS Analysis

Chromatographic separation was performed on a Kinetex C18 column (100 Å, 2.6 μm, 100 mm × 2.1 mm i.d.) maintained at 40 °C. The mobile phase consisted of (A) 5 mmol/L ammonium acetate in water and (B) acetonitrile. The gradient program was the following: 0–2 min, 10–60% B; 2–9 min, 60–80% B; 9–10 min, 80–100% B; 10–12 min, 100% B; 12–14 min, 100–10% B; 14–20 min, 10% B. The flow rate was 0.3 mL/min, and the injection volume was 1 μL. Mass spectrometric detection was performed using electrospray ionization (ESI) in negative ion mode with multiple reaction monitoring (MRM). The optimized source parameters were Curtain Gas (CUR): 35 psi; IonSpray Voltage (IS): −4500 V; Temperature (TEM): 550 °C; Ion Source Gas 1 (GS1): 55 psi; Ion Source Gas 2 (GS2): 60 psi [49].

### 3.5. Migration Studies

#### 3.5.1. Migration Tests Under Intended Use Conditions

Three pieces (1 cm × 1 cm) of each sample were immersed in 10 mL of food simulant within a 20 mL PP centrifuge tube (surface-to-volume ratio S/V ≈ 6 dm^2^/L). The simulants used were the following: ultrapure water (aqueous foods), 4% (*v*/*v*) acetic acid (acidic foods), 50% (*v*/*v*) ethanol (alcoholic foods), and 95% (*v*/*v*) ethanol (fatty foods). Migration conditions (time, temperature) were selected based on the intended use of the tableware (containers or cups) and the simulant type, as detailed in Table 6, following principles outlined in the literature [16,50]. After migration, 1 mL of simulant was filtered (0.22 μm nylon) into an LC vial for analysis. Triplicate migrations and simulant blanks were performed.

#### 3.5.2. Impact of Simulated Takeout Conditions on Migration

Given that PFOA showed the highest residue levels and occasional exceedances, its migration under simulated takeout scenarios was investigated using the simulant showing the highest migration (95% ethanol) and a sample (T2) exceeding the PFOA residue limit. The experimental design steps are as follows:

Control (Static Migration): Samples were immersed in 95% ethanol (S/V = 6 dm^2^/L) and held statically in a 50 °C water bath for 1, 2, 4, 6, 8, 10, and 12 h.

Oscillation (Simulated Transport): Samples were immersed in 95% ethanol (S/V = 6 dm^2^/L) and oscillated (120 rpm) in a 50 °C water bath for 1, 2, 4, 6, 8, 10, and 12 h.

Post-Oscillation Storage: After 1 h of oscillation (simulating typical delivery time), samples were stored under two conditions, as follows.

Refrigerated Storage (4 °C): Stored in a closed refrigerator. The simulant was sampled every 2 h (total storage: 0, 2, 4, 6, 8, 10, 12 h). The refrigerator door was only opened briefly for sampling.

Room Temperature Storage (25 °C): Stored at ambient temperature. The simulant was sampled at 0, 2, 4, 6, 8, 10, and 12 h.

Simulated Reheating: As microwave-heating organic solvents is unsafe, reheating was simulated by immersing the sample (after storage) in fresh 60 °C ultrapure water for 15 min (S/V = 6 dm^2^/L).

After each step, 1 mL of simulant was filtered (0.22 μm nylon) into an LC vial for PFOA analysis by LC-MS/MS. Triplicate tests and blanks were performed.

#### 3.5.3. Calculation of Residues and Migration

$$\mathrm{PFAS}\;\mathrm{Residue}\;(X_{1},\;\mathrm{mg}/\mathrm{kg}):\;X_{1}=\frac{C\times V}{m}$$

where *C* = concentration in final extract determined by LC-MS/MS (mg/L); *V* = volume of final extract (L); *m* = sample mass (kg).

$$\mathrm{Specific}\;\mathrm{Migration}\;(X_{2},\;\mathrm{mg}/\mathrm{kg}):\;X_{2}=\frac{\rho \times V}{m}$$

where *ρ* = concentration in food simulant determined by LC-MS/MS (mg/L); *V* = volume of food simulant (L); *m* = mass of food simulant (kg). (Note: Density of simulants approximated as 1 g/mL for conversion to mass).

### 3.6. Data Analysis

LC-MS/MS data were processed using SCIEX OS software (Version 1.4). Statistical analysis (one-way ANOVA) and graphical visualization were performed using SPSS software (Version 20, IBM Corp., Armonk, NY, USA) and OriginPro 2020 (OriginLab Corp., Northampton, MA, USA), respectively. The data are expressed as the mean ± expanded uncertainty. A significance level of *p* < 0.05 was used.

## 4. Conclusions

This study provides a comprehensive safety assessment of PFASs in commercial sugarcane pulp tableware, integrating residue analysis and migration kinetics under simulated use conditions. Despite elevated levels of total fluorine (TF > 50 mg/kg in 31% of samples) and sporadic PFOA residues exceeding EU limits (25 μg/kg in 9% of samples), the migration of five target PFASs (PFOA, PFOS, PFNA, PFHxA, PFPeA) from sugarcane pulp tableware into food simulants (water, 4% HAc, 50% EtOH, 95% EtOH) remained exceptionally low (≤0.70 μg/kg) under standardized conditions and minimally increased to 0.99 μg/kg under aggressive simulated takeout scenarios (50 °C oscillation + 25 °C storage). Crucially, all migrated levels were >15-fold below the 0.01 mg/kg safety threshold, with low extractable organic fluorine (EOF/TF: 3.19–10.4%) confirming inorganic fluorides as primary TF contributors. These results indicate negligible consumer exposure risk during typical use but warrant stricter manufacturing controls targeting PFOA contamination sources and inorganic fluoride inputs. The results support EU PPWD limits. The U.S. FDA may consider TF monitoring for plant-based FCMs. Future studies should (1) assess long-term migration under repeated use; (2) employ FTIR to characterize structural changes during migration; (3) investigate precursor transformation pathways; and (4) evaluate cyclic exposure (e.g., 10× reuse with acidic food).

## Figures and Tables

**Table 1:** Linear range, LOD, LOQ, linear equation, and R^2^ for TF and EOF determination by ISE.

Analyte	Linear Range (μg/L)	LOD (μg/L)	LOQ (μg/L)	Linear Equation	R^2^
TF	6–100	3.0	6.0	Y = 407.76 − 53.03X	0.9998
EOF	4–100	2.0	4.0	Y = 407.76 − 53.03X	0.9998

**Table 2:** Average spike recovery and precision (RSD, *n* = 6) for TF and EOF.

Analyte	Spike Level (μg/L)	Avg. Recovery (%)
TF	10.0	87.1 ± 6.5
	40.0	102.6 ± 4.6
	80.0	111.4 ± 7.9
EOF	10.0	103.8 ± 6.7
	40.0	97.5 ± 2.2
	80.0	108.7 ± 3.8

**Table 3:** Validation parameters for LC-MS/MS analysis of PFAS residues.

PFAS	Linear Range (μg/L)	LOD (μg/L)	LOQ (μg/L)	Linear Equation	R^2^
PFOA	0.5–40	0.2	0.5	y = 4568.7x + 1973.9	0.9999
PFOS	1.2–40	0.5	1.2	y = 16,035.1x + 298.5	0.9999
PFNA	0.5–40	0.2	0.5	y = 20,900.7x + 773.8	0.9998
PFHxA	0.5–40	0.2	0.5	y = 33,342.9x + 5325.6	0.9998
PFPeA	0.5–40	0.2	0.5	y = 62,119.2x + 5372.3	0.9998

**Table 4:** Average spike recovery for PFAS residues in methanol and low-PFAS matrix.

Substance	Spiked Level (μg/L)	Average Recovery (%)				
		Methanol	Ultrapure Water	4% Acetic Acid	50% Ethanol	95% Ethanol
PFOA	5.0	86.2 ± 8.9	115.4 ± 8.1	98.3 ± 7.2	114.3 ± 6.2	103.4 ± 4.8
	7.5	107.4 ± 7.1	83.8 ± 3.1	109.1 ± 3.9	110.1 ± 5.5	88.5 ± 5.0
	30.0	93.1 ± 7.2	100.7 ± 0.8	90.0 ± 6.5	105.4 ± 6.2	101.9 ± 6.8
PFOS	5.0	89.7 ± 3.17	105.4 ± 7.3	89.5 ± 5.5	105.8 ± 7.1	96.4 ± 7.0
	7.5	95.6 ± 3.4	108.8 ± 6.4	99.7 ± 5.2	101.0 ± 5.2	97.7 ± 6.3
	30	93.8 ± 7.1	110.7 ± 4.9	104.1 ± 7.3	99.3 ± 5.0	89.7 ± 5.2
PFNA	5.0	85.9 ± 1.5	92.3 ± 7.3	87.2 ± 3.2	98.3 ± 6.7	97.5 ± 6.1
	7.5	95.9 ± 2.9	104.8 ± 4.3	98.3 ± 0.8	86.8 ± 2.9	88.9 ± 4.5
	30.0	104.6 ± 6.5	92.9 ± 2.3	104.3 ± 1.2	95.4 ± 4.2	93.4 ± 7.3
PFHxA	5.0	91.7 ± 8.9	112.5 ± 3.2	96.3 ± 0.7	105.9 ± 3.2	93.4 ± 2.8
	7.5	96.7 ± 3.8	107.1 ± 1.4	100.5 ± 3.9	99.3 ± 3.1	91.8 ± 3.7
	30.0	99.4 ± 6.8	100.7 ± 0.8	98.0 ± 5.5	95.3 ± 2.9	94.5 ± 4.5
PFPeA	5.0	90.9 ± 7.4	112.1 ± 8.8	96.5 ± 0.8	101.0 ± 4.8	98.1 ± 2.9
	7.5	99.6 ± 6.8	118.6 ± 1.4	94.5 ± 2.3	99.1 ± 5.3	96.8 ± 3.5
	30.0	97.0 ± 6.1	108.7 ± 5.4	92.1 ± 8.2	103.2 ± 5.9	87.8 ± 2.4

**Table 5:** Validation parameters (linear range, LOD, LOQ, R^2^) for LC-MS/MS analysis of PFAS migration into different food simulants.

Name	Food Simulant	Linear Equation	R^2^	Linear Range (μg/L)	LOD/(μg/L)	LOQ/(μg/L)
PFOA	Ultrapure water	Y = 35,311.9x + 201,942.6	0.9999	0.5~40	0.2	0.5
	4% acetic acid	Y = 39,154.6x + 265,547.2	0.9997	0.5~40	0.2	0.5
	50% ethanol	Y = 51,231.5x + 269,475.3	0.9987	0.5~40	0.2	0.5
	95% ethanol	Y = 24,186.9x + 289,795.3	0.9986	0.5~40	0.2	0.5
PFOS	Ultrapure water	Y = 2572.5x + 2772.1	0.9999	1.5~40	0.5	1.5
	4% acetic acid	Y = 2258.7x + 711.1	0.9971	3.0~40	0.9	3.0
	50% ethanol	Y = 4587.1x + 1028	0.9998	0.8~40	0.3	0.8
	95% ethanol	Y = 1353x + 4179.1	0.9991	3.0~40	0.9	3.0
PFNA	Ultrapure water	Y = 24,917.4x − 10,909.3	0.9984	0.5~40	0.1	0.3
	4% acetic acid	Y = 20,301.5x − 1033.1	0.9979	0.5~40	0.2	0.5
	50% ethanol	Y = 35,932.5x + 6584.2	0.9999	0.5~40	0.1	0.3
	95% ethanol	Y = 16,679.2x + 37,326.8	0.9977	0.5~40	0.2	0.5
PFHxA	Ultrapure water	Y = 2250.2x − 387.9	0.9981	0.5~40	0.2	0.5
	4% acetic acid	Y = 2594.4x + 485.1	0.9984	0.5~40	0.2	0.5
	50% ethanol	Y = 2639.7x + 835.1	0.9999	0.5~40	0.2	0.5
	95% ethanol	Y = 1249.12x + 7812	0.9999	0.5~40	0.2	0.5
PFPeA	Ultrapure water	Y = 34,432.7x + 12,957.1	0.9993	0.5~40	0.2	0.5
	4% acetic acid	Y = 39,984.1x + 38,144.2	0.9995	0.5~40	0.2	0.5
	50% ethanol	Y = 41,437.8x + 16,946	0.9999	0.5~40	0.2	0.5
	95% ethanol	Y = 23,838.6x + 83,774.2	0.9999	0.5~40	0.2	0.5

**Table 6:** Migration test conditions for sugarcane pulp food containers and tea cups.

Sample Type	Expected Contact Time	Food Simulants	Migration Conditions
T1–T13 (Containers)	60 < t ≤ 90 min	50% EtOH, 95% EtOH	Reflux Temp, 2 h
		Ultrapure H_2_O, 4% HAc	100 °C, 2 h
C1–C9 (Tea Cups)	5 < t ≤ 30 min	50% EtOH, 95% EtOH	Reflux Temp, 30 min
		Ultrapure H_2_O, 4% HAc	100 °C, 30 min

Reflux temperature for the respective ethanol solution (approx. 82 °C for 50% EtOH, 78 °C for 95% EtOH). Measured reflux temps: 50% EtOH = 82.3 ± 0.5 °C; 95% EtOH = 78.2 ± 0.3 °C. Note: For tea cups coming into contact with boiling water, migration was conducted in ultrapure water.

**Table 7:** Source information regarding studied sugarcane pulp tableware samples.

Sample ID	Max. Use Temp. (°C)	Microwave Safe	Sample ID	Max. Use Temp. (°C)	Microwave Safe
T1	120	Yes	T12	120	Yes
T2	120	Yes	T13	120	Yes
T3	170	Yes	C1	120	N/A
T4	120	Yes	C2	120	N/A
T5	120	Yes	C3	100	N/A
T6	150	Yes	C4	100	N/A
T7	100	Yes	C5	100	N/A
T8	120	Yes	C6	100	N/A
T9	120	Yes	C7	120	N/A
T10	120	Yes	C8	120	N/A
T11	120	Yes	C9	120	N/A

N/A: Information not provided by the manufacturer/seller.

## Data Availability

The original contributions presented in this study are included in the article/Supplementary Materials. Further inquiries can be directed to the corresponding authors.

## Supplementary Materials

The following supporting information can be downloaded at https://www.mdpi.com/article/10.3390/molecules30153166/s1, Figure S1: Total ion chromatograms of five PFAS reference standards in LC-MS/MS.
