# Supplementary material for: Migration Safety of Perfluoroalkyl Substances from Sugarcane Pulp Tableware: Residue Analysis and Takeout Simulation Study
Source: Molecules. 2025 Jul 29;30(15):3166. doi: 10.3390/molecules30153166 (PMC12348458; doi:10.3390/molecules30153166)

LC-MS/MS chromatograms and calibration curves

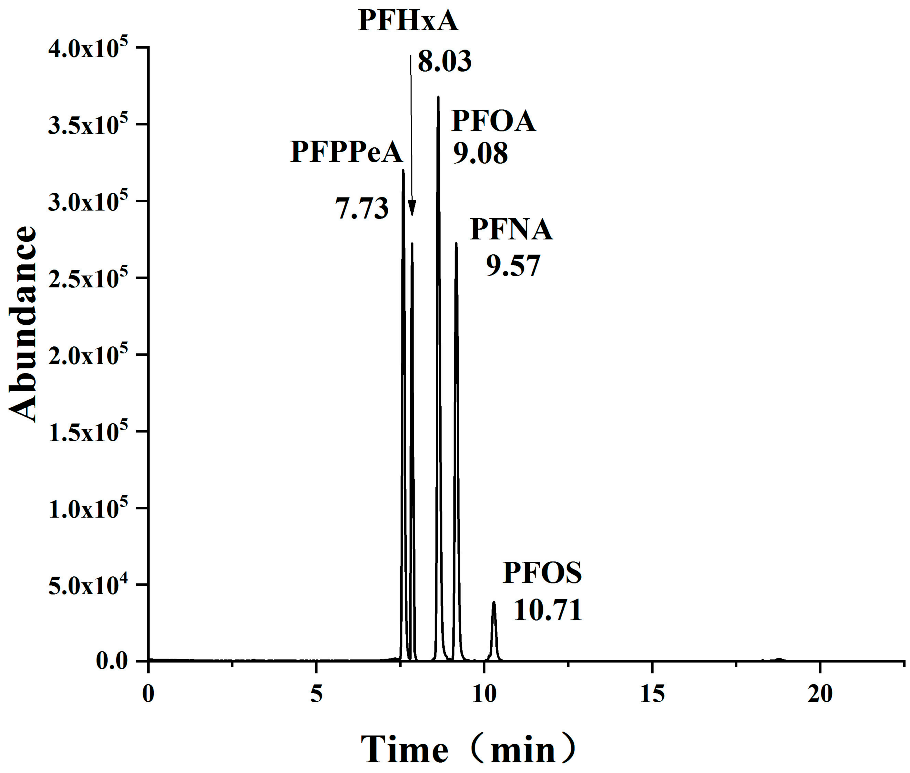

Figure S1. Total ion chromatograms of five PFAS reference standards in LC-MS/MS

pfna

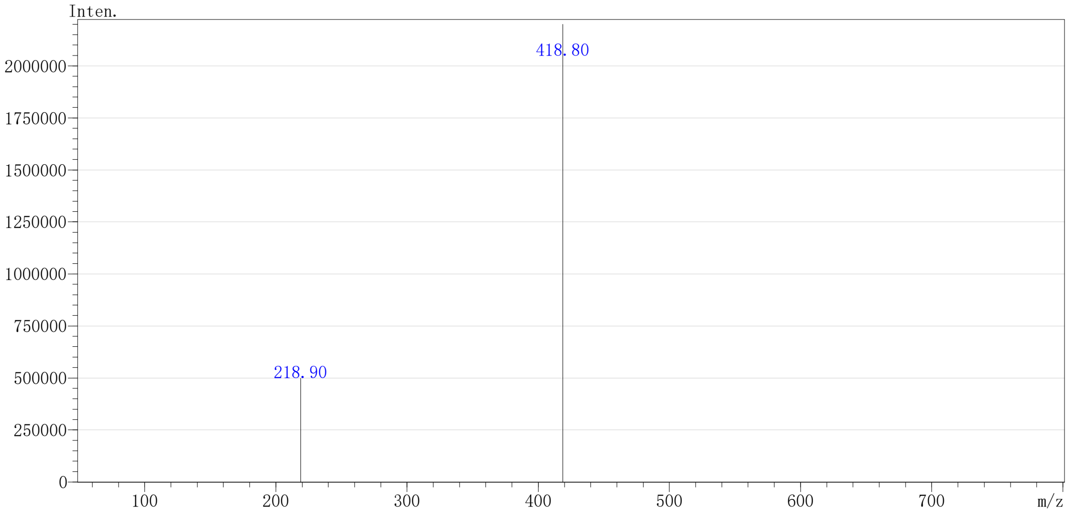

Pfhxa

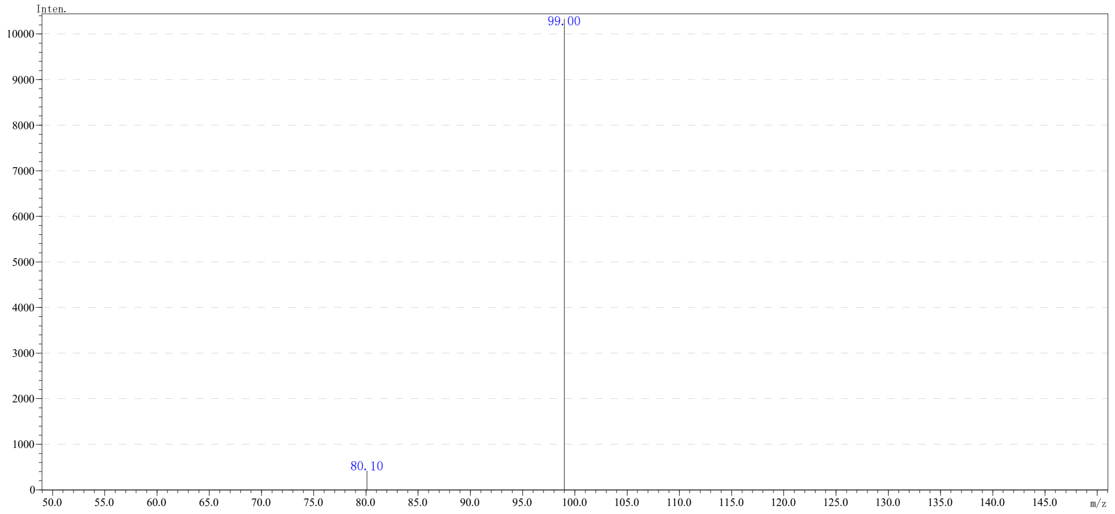

PFOA

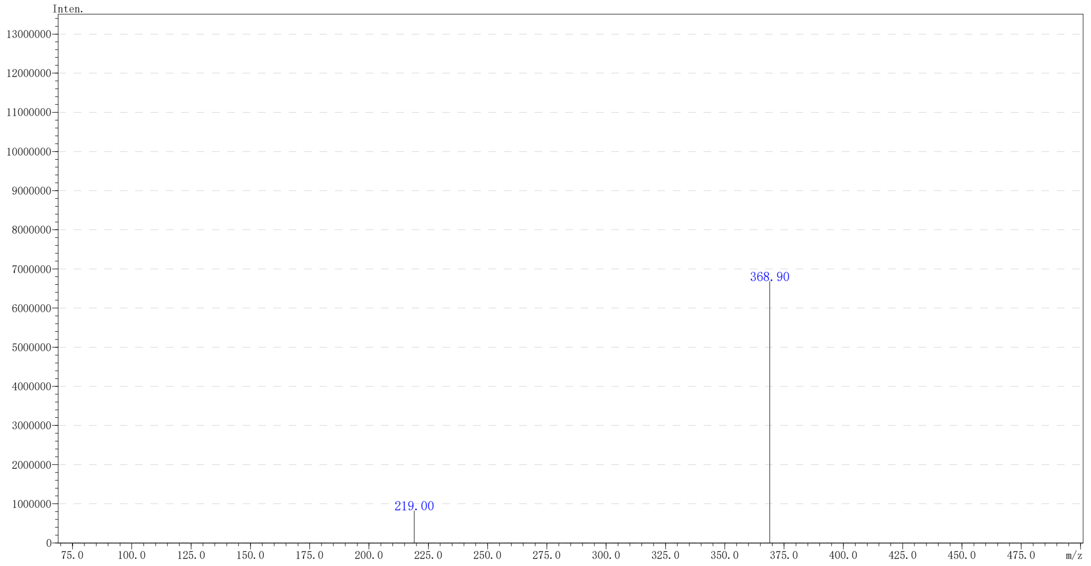

Pfpea

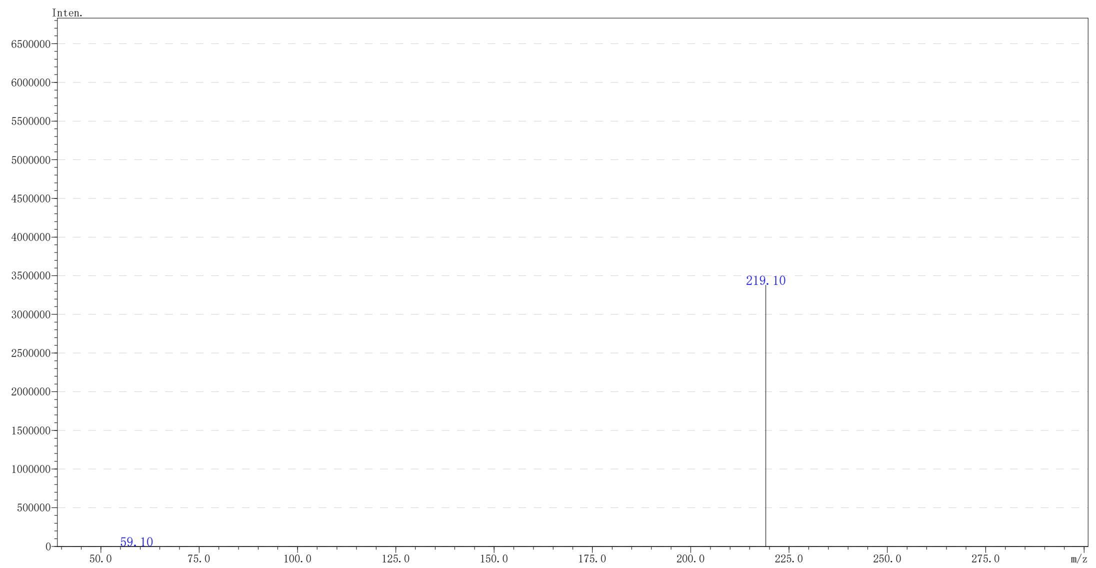

Pfos

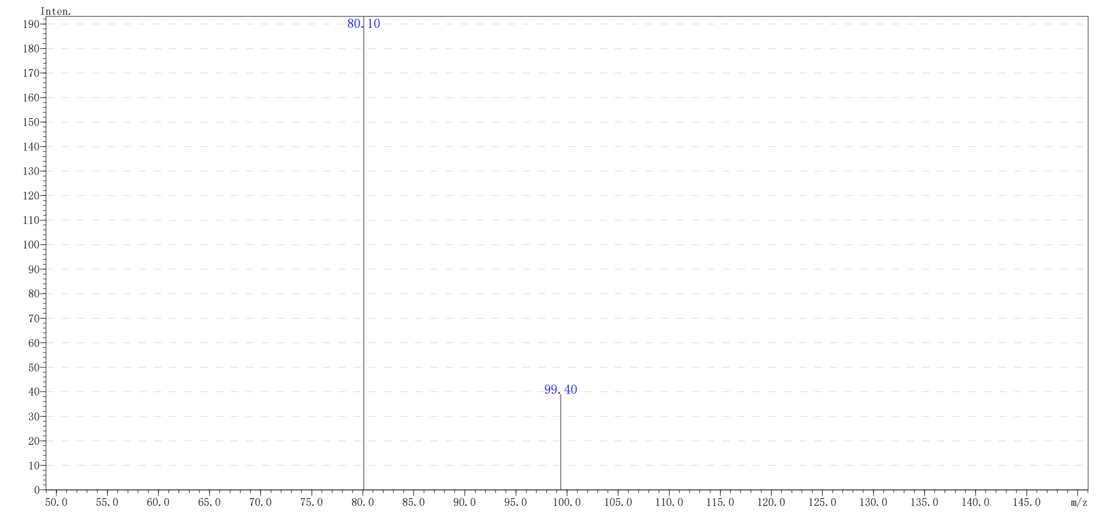

Supplement: Supplementary file 1 [file molecules-30-03166-s001.zip › molecules-3764768-supplementary.pdf]
